# Comparative genomic analysis and characteristics of NCCP15740, the major type of enterotoxigenic *Escherichia coli* in Korea

**DOI:** 10.1186/s13099-017-0204-y

**Published:** 2017-09-20

**Authors:** Taesoo Kwon, Si-yun Chung, Young-Hee Jung, Su-Jin Jung, Sang-Gyun Roh, Je-Seop Park, Cheorl-Ho Kim, Won Kim, Young-Seok Bak, Seung-Hak Cho

**Affiliations:** 1 Interdisciplinary Program in Bioinformatics, Seoul National University, 1 Gwanak-ro, Gwanak-gu, Seoul, 151-742 Republic of Korea; 2 Department of Biotechnology, Korea University, Anam-ro 145, Seongbuk-gu, Seoul, 02841 Republic of Korea; 3 Department of Dental Hygiene, College of Medical Science, Konyang University, 158, Gwanjeodong-ro, Seo-gu, Daejeon-Metropolitan City, 35365 Republic of Korea; 4 Department of Emergency Medical Services, Sun Moon University, Asan, Chungcheongnam-do 31460 Republic of Korea; 5 Fire Science Laboratory, National Fire Service Academy, Asan, Chungcheongnam-do 31555 Republic of Korea; 6 Glycobiology Unit, Department of Biological Science, Sungkyunkwan University and Samsung Advanced Institute for Health Sciences and Technology (SAIHST), 2066 Seobu-ro, Suwon, 16419 Republic of Korea; 7 Division of Enteric Diseases, Center for Infectious Diseases, Korea National Institute of Health, Cheongju, 363-951 Republic of Korea

**Keywords:** Enterotoxigenic *Escherichia coli* O6, Whole-genome sequencing, Colonization factor genes, Enterotoxin, Multilocus sequence typing, Virulence

## Abstract

**Background:**

Enterotoxigenic *Escherichia coli* (ETEC) cause infectious diarrhea and diarrheal death. However, the genetic properties of pathogenic strains vary spatially and temporally, making prevention and treatment difficult. In this study, the genomic features of the major type of ETEC in Korea from 2003 to 2011 were examined by whole-genome sequencing of strain NCCP15740, and a comparative genomic analysis was performed with O6 reference strains.

**Results:**

The assembled genome size of NCCP15740 was 4,795,873 bp with 50.54% G+C content. Using rapid annotation using subsystem technology analysis, we predicted 4492 ORFs and 17 RNA genes. NCCP15740 was investigated for enterotoxin genes, colonization factor (CF) genes, serotype, multilocus sequence typing (MLST) profiles, and classical and nonclassical virulence factors. NCCP15740 belonged to the O6:H16 serotype and possessed enterotoxin genes encoding heat-stable toxin (STh) and heat-labile toxin (LT); 87.5% of the O6 serotype strains possessed both toxin types. NCCP15740 carried the colonization factors CS2 and CS3, whereas most O6 strains carried CS2-CS3-CS21 (79.2%). NCCP15740 harbored fewer virulence factors (59.4%) than the average observed in other O6 strains (62.0%). Interestingly, NCCP15740 did not harbor any nonclassical virulence genes.

**Conclusions:**

The major type of ETEC in Korea had the same MLST sequence type as that of isolates from the USA obtained in 2011 and 2014, but had different colonization factor types and virulence profiles. These results provide important information for the development of an ETEC vaccine candidate.

**Electronic supplementary material:**

The online version of this article (doi:10.1186/s13099-017-0204-y) contains supplementary material, which is available to authorized users.

## Background

*Escherichia coli* is a rod-shaped, gram-negative, facultative anaerobic and non sporulating bacterium belonging to the family Enterobacteriaceae. *E. coli* inhabits the intestines of all humans and animals. Most *E. coli* are harmless, but some induce various diseases; thus, the species is considered an opportunistic pathogen. *E. coli* strains that cause diarrhea can be categorized into six groups according to virulence elements in the genome: enterotoxigenic (ETEC), enteropathogenic, nonsporulating, enteroaggregative, enteroinvasive, and diffusely adherent [1]. ETEC is a major cause of traveler’s diarrhea and is responsible for 700,000 diarrhea-related deaths per year in young children of less than 5 years of age in developing countries [2, 3]. Among the major virulence factors, two enterotoxins, i.e., a heat-labile toxin (LT) and a heat-stable toxin (ST), induce watery diarrhea in ETEC. The LT toxin is encoded by the *eltAB* gene. ST toxins are classified into two types, STh and STp; human-derived STh is encoded by *estA*, and porcine-derived STp is encoded by *st1* [4]. In addition to serotyping, ETEC strains are classified by the combination of the O antigen of the lipopolysaccharide, H antigen of the flagellin, and K antigens. Although there are over 100 different O antigens and 34 H antigens associated with ETEC [5, 6], O6, O8, O25, O78, O128, and O153 and H7, H12, H16, H21, H45, and H49 are the most common, respectively [7]. In addition to enterotoxins, ETEC strains possess adhesive pili called colonization factors (CFs), which mediate adherence to the small intestinal wall. Over 30 CFs have been described in human ETEC strains to date. The most prevalent CFs are CFA/I and CS1–CS6, and strains typically carry two or three CFs, such as CS1 + CS3, CS2 + CS3, and CS5 + CS6.

In a previous study [8], 258 isolates from patients with diarrhea in Korea and 33 isolates from travelers visiting other Asian countries were analyzed, and two major sequence types were identified by multilocus sequence typing (MLST). In particular, ST171 (n = 62) was identified as the most prevalent ETEC type in Korea, but ST949 (n = 5) was the most frequent among inflow isolates. Although ST171 was a major MLST type of ETEC in Korea, the genomic characteristics, including enterotoxin genes, CF genes, and virulence factors, had not yet been investigated. In the present study, we selected one ST171 strain identified in this previous work, i.e., NCCP15740, isolated in 2010 from a patient with diarrhea, with serotype O6:H16, and performed whole-genome sequencing. We compared the genome of NCCP15740 with other whole-genome sequences of ETEC strains reported as O6:H16 isolates over a similar time period.

## Methods

### Strains, isolation, and serotyping

*Escherichia coli* NCCP15740 was isolated in 2010 from a patient with diarrhea and identified as a major MLST type (ST 171) of ETEC in Korea based on 24 isolates obtained from 2003 to 2011 [8]. Candidate colonies of NCCP15740 were identified based on phenotypes and biochemical properties using the API20E system (Biomerieux, Marcy l’Etoile, France). *E. coli* ATCC 25922 [9] was used as a reference strain to investigate the characteristics of NCCP15740. *E. coli* ATCC 25922 is an O6 serotype ETEC (O6:H1) reference strain. Moreover, we selected 19 *E. coli* O6 strains (O6:H16) [10, 11] as reference strains because they had the same serotype as NCCP15740. The 19 *E. coli* O6 strains were isolated in the USA from 2011 to 2014. From the comparison with the 19 *E. coli* O6 strains, we expected that the evolutionary relationship with the strains identified from a similar period as NCCP15740 could be estimated. Two additional strains were used as reference strains: *E. coli* O6:H16:CFA/II str. B2C (traveler’s diarrhea) [12] and *E. coli* O6:H16 str. 99-3165 (USA) [13].

### Library preparation and whole-genome sequencing

A TruSeq sample preparation kit (Illumina, San Diego, CA, USA) was used to construct a sequencing library. Whole-genome sequencing of NCCP15740 was performed using the Illumina HiSeq 2000 platform (Theragen Etex Bio Institute, Suwon, Republic of Korea).

### Genome assembly and annotation

High-quality reads were obtained by discarding reads with quality scores of less than Q20 and were assembled into scaffolds, using SOAPdenovo (version 1.05) [14]. Open reading frames were predicted and annotated by rapid annotation using subsystem technology (RAST, version 4.0) server [15]. In silico serotyping of NCCP15740 and other reference strains was performed using SerotypeFinder (version 1.1) [16]. MLST typing was also performed using the *E. coli* MLST database [17]. The genomic and phenotypic characteristics of NCCP15740 and the reference strains are summarized in Table 1.

**Table 1:** Genomic features of NCCP15740

Strain	NCCP15740
Genome (Mb)	4.79
%GC	50.54
Total open reading frames	4492
tRNAs	17
rRNAs	0

### Phylogenetic analysis

Multiple sequence alignments were obtained from whole-genome sequences of 24 *E. coli* strains and from seven MLST genes of the *E. coli* isolates, i.e. *adk*, *fumC*, *gyrB*, *icd*, *mdh*, *purA*, and *recA* [18, 19], using Mugsy (version 1.2.3) [20]. Approximate maximum-likelihood phylogenetic trees were generated using FastTree (version 2.1.7) [21] with the generalized time-reversible + CAT model [22]. The resulting trees were visualized using FigTree (version 1.3.1; http://tree.bio.ed.ac.uk/software/figtree/).

### Analysis of virulence factors

To inspect virulence factor-encoding genes, BLAST searches of whole coding sequences (CDSs) were performed against the virulence factor database VFDB [23] adopting an e-value threshold of 1e-5. In addition, the BLAST Score Ratio (BSR) [24] was calculated to identify homologous virulence factor genes. A BSR threshold of at least 0.7 was used in this study.

### Quality assurance

The genomic DNAs were purified from a pure culture of a single bacterial isolate of NCCP15740. Potential contamination of the genomic libraries by other microorganisms was evaluated using a BLAST search against the nonredundant database.

## Results

### General features

**Fig. 1:**
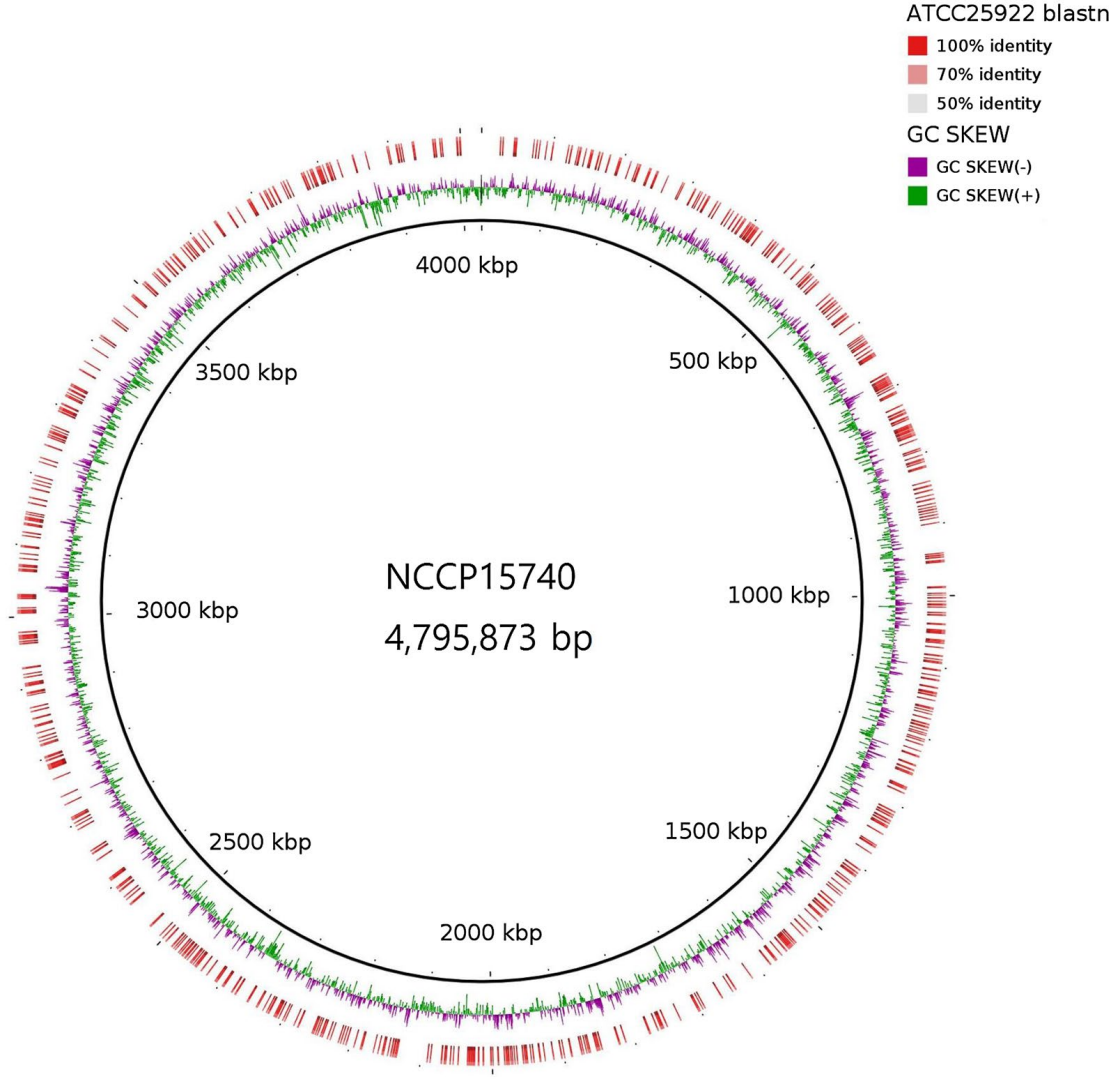
Circular map of NCCP15740. The map was created using BLAST ring image generator (BRIG) version 0.95

Using the Illumina HiSeq 2000 platform, we generated a total of 548,710,000 bp paired-end reads (86.32-fold coverage). After quality control, 495 Mbp of high-quality reads were de novo assembled into 156 scaffolds with a scaffold N50 of 87,362 bp. The NCCP15740 genome was 4,795,873 bp in length (Fig. 1), and the G+C content was 50.54%. Using the RAST server pipeline, 4492 putative coding sequences and 17 RNA genes were identified. The genomic properties of NCCP15740 are summarized in Table 1. According to in silico analysis, the NCCP15740 serotype was O6:H16.

### Phylogenetic analysis

**Fig. 2:**
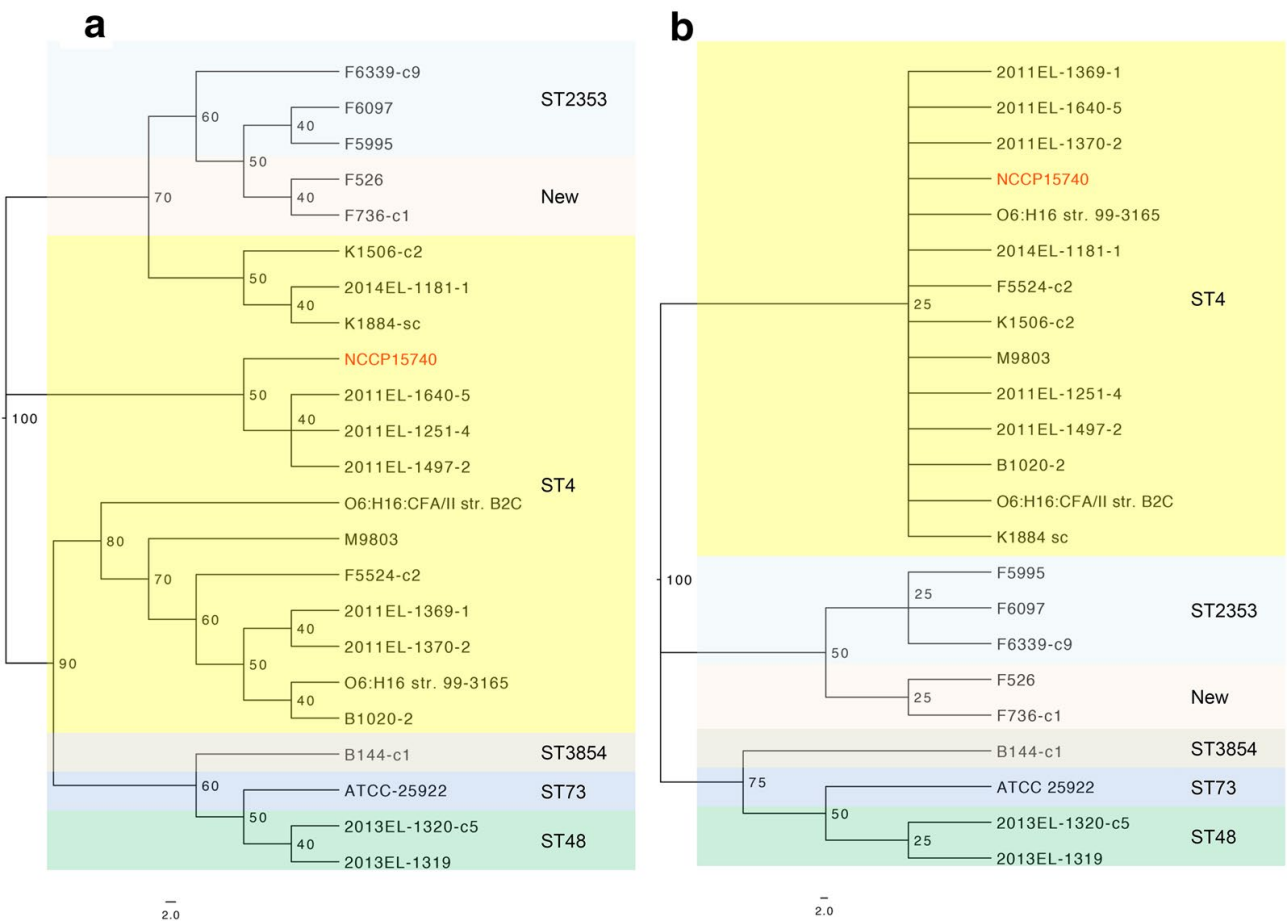
Phylogenetic tree of NCCP15740. **a** Whole-genome phylogeny, **b** MLST-based phylogeny. Evolutionary time is scaled by 100; lower values imply a relatively recent branching event. The scale bar indicates 2.0 substitutions per site. NCCP15740 (red) was placed in a single clade with 2011EL 1251-4, 2011EL 1497-2, and 2011EL 1640-5 in both the whole-genome phylogeny and the MLST phylogeny

A whole-genome phylogeny was constructed from the alignments of the genomes of 24 *E. coli* isolates, and an MLST-based phylogeny was constructed from the alignments of seven MLST genes of the *E. coli* isolates (Fig. 2). The *E. coli* O6 strains had simple phylogenetic relationships, represented by three sequence types, according to both the whole-genome and MLST data. The whole-genome phylogeny showed that NCCP15740 belonged to a group of strains isolated in 2011 and was distinct from the majority of O6 strains, although it was isolated in 2010. In contrast to the whole-genome phylogeny, all isolates obtained in 2011 clustered in the same group in the MLST-based phylogeny. Only three 2011 isolates clustered with NCCP15740 in the whole-genome phylogeny, whereas 14 isolates obtained in 2011 formed a cluster in the MLST-based phylogeny. Based on MLST, the most prevalent sequence type was ST4 (62.5%), followed by ST2353 (12.5%). The sequence type of NCCP15740 was ST4.

### Identification of enterotoxins and colonization surface antigens

We investigated the toxin types of NCCP15740 and reference strains. As shown in Table 2, consistent with the major type of Korean ETEC isolates, NCCP15740 carried both LT and ST enterotoxins, and the presence of both STh and LT was common in the ETEC O6 strains (21/24). Moreover, CS types were found in 87.5% of all isolates, including NCCP15740. Among the strains of O6 serotype in this study, CS2 + CS3 + CS21 was the most prevalent type, and four different CS types were found. CS21 was detected in 20 (83.3%) of the 24 ETEC O6 strains, but was not detected in two (8.3%) strains containing only STh and in one (4.1%) strain containing both STh and LT, i.e., NCCP15740.

**Table 2:** Genomic features of NCCP15740 and other strains used in this study

Strain	Genome (Mb)	MLST type	Serotype	Toxin type	CS type	GenBank accession	GenBank assembly
*E. coli* NCCP15740	4.79	ST4	O6:H16	STh + LT	CS2 + CS3	MOED01	GCA_001865725
*E. coli* ATCC25922	5.20	ST73	O6:H1	None	None	CP009072	GCA_000743255
*E. coli* O6:H16:CFA/II str. B2C	5.02	ST4	O6:H16	STh + LT	CS2 + CS3 + CS21	AUZS01	GCA_000517245
*E. coli* O6:H16 str. 99-3165	4.75	ST4	O6:H16	STh + LT	CS2 + CS3 + CS21	JHJW01	GCA_000614435
*E. coli* strain 2011EL-1370-2	4.81	ST4	O6:H16	STh + LT	CS2 + CS3 + CS21	JPUT01	GCA_000770035
*E. coli* strain 2011EL-1369-1	4.80	ST4	O6:H16	STh + LT	CS2 + CS3 + CS21	JPUS01	GCA_000770045
*E. coli* strain 2011EL-1497-2	4.97	ST4	O6:H16	STh + LT	CS2 + CS3 + CS21	JPUU01	GCA_000770055
*E. coli* strain 2013EL-1320-c5	4.71	ST48	O6:H16	STh	None	JPUD01	GCA_000770255
*E. coli* strain 2013EL-1319	4.71	ST48	O6:H16	STh	None	JPIF01	GCA_000773435
*E. coli* strain F5995	5.17	ST2353	O6:H16	STh + LT	CS2 + CS3 + CS21	JPXL01	GCA_000773475
*E. coli* strain M9803	4.88	ST4	O6:H16	STh + LT	CS2 + CS3 + CS21	JPXK01	GCA_000773535
*E. coli* strain F6097	5.19	ST2353	O6:H16	STh + LT	CS2 + CS3 + CS21	JPXJ01	GCA_000773555
*E. coli* strain 2011EL-1640-5	4.87	ST4	O6:H16	STh + LT	CS2 + CS3 + CS21	JPXN01	GCA_000773575
*E. coli* strain 2011EL-1251-4	4.88	ST4	O6:H16	STh + LT	CS2 + CS3 + CS21	JPXM01	GCA_000773595
*E. coli* strain K1506-c2	4.94	ST4	O6:H16	STh + LT	CS2 + CS3 + CS21	JYIB01	GCA_000935345
*E. coli* strain F526	4.95	New	O6:H16	STh + LT	CS2 + CS3 + CS21	JYHX01	GCA_000935365
*E. coli* strain 2014EL-1181-1	4.95	ST4	O6:H16	STh + LT	CS2 + CS3 + CS21	JYIE01	GCA_000935375
*E. coli* strain F736-c1	4.95	New	O6:H16	STh + LT	CS2 + CS3 + CS21	JYHY01	GCA_000935425
*E. coli* strain B144-c1	4.90	ST3854	O6:H16	STh + LT	CS2 + CS3 + CS21	JYID01	GCA_000935435
*E. coli* strain K1884 sc	4.99	ST4	O6:H16	STh + LT	CS2 + CS3 + CS21	JYIC01	GCA_000935445
*E. coli* strain B1020-2	4.77	ST4	O6:H16	STh + LT	CS2 + CS3 + CS21	JYIF01	GCA_000935515
*E. coli* strain F6339-c9	4.91	ST2353	O6:H16	STh + LT	CS2 + CS3 + CS21	JYIH01	GCA_000935525
*E. coli* strain F5524-c2	4.88	ST4	O6:H16	STh + LT	CS2 + CS3 + CS21	JYIG01	GCA_000935555

### Analysis of virulence factors

To determine the causal mechanisms underlying the observed pathogenicity [8], we compared virulence factors in NCCP15740 with those of the reference strains (Additional file 1: Figure S1). The strains harbored 207 total virulence factors classified into 27 categories and 66 subcategories. NCCP15740 harbored 123 of the 207 virulence factors (59.4%), which was fewer than the average number of virulence factors in the reference strains used in this study (128/207, 62.0%). Several virulence factors that were found in the majority of the O6 strains were not present in NCCP15740, including *ibeB*, *etpA*, *cah*, *fimZ*, *tia*, *tuf*, *flgD*, *flgE*, *ipaH2.5*, and *aatC*. Nonclassical virulence factors related to adherence, invasion, secretion, and iron acquisition are the main contributors to ETEC diarrhea [25]. Surprisingly, most of the nonclassical virulence factors that have been found in ETEC strains [25], including *eatA*, *etpB*, *fyuA*, *leoA*, and *tibA*, were not present in O6 strains. Only three nonclassical virulence factors were found in O6 strains, i.e., *etpA* (18 out of 24), *irp2* (only in *E. coli* ATCC 25922), and *tia* (11 out of 24). However, none of the nonclassical virulence factors were found in NCCP15740.

## Discussion

ETEC is responsible for 700,000 diarrhea-related deaths per year in young children of less than 5 years of age and is a main cause of traveler’s diarrhea [3]. However, the type and relative proportions of ETEC enterotoxins differ depending on the geographical source.

The enterotoxin types of Korean isolates from 2003 to 2011 were reported to be similar to those of isolates from Asia and the Middle East, but different from those of isolates from South America [8]. In this study, we investigated the characteristics of the major type of ETEC in Korea at the genomic level by sequencing an ST171 isolate, NCCP15740, and performing a comparative analysis with the genome sequences of other O6 strains. According to the whole-genome phylogeny, NCCP15740 belonged to one of the two groups of strains that were isolated in 2011, but belonged to the group that included the majority of O6 strains in the MLST-based phylogeny (Fig. 2). There are many genomic changes that determine the branch of a strain in a phylogenetic tree, including SNPs, insertions, deletions, prophages, and other insertion sequence elements. However, MLST genes are housekeeping genes and are more conserved than other genomic loci. Therefore, whole-genome-based phylogeny is more sensitive than MLST-based phylogeny, although it is more difficult to group strains with whole-genome-based phylogeny. Accordingly, it is necessary to select whole-genome- or MLST-based phylogeny according to the needs of the study design and aim. An MLST-base phylogeny is suitable for clustering strains according to their MLST type, whereas whole-genome phylogeny provides a better representation of the differences between strains than MLST-based phylogeny.

We investigated the toxin types of NCCP15740 and reference strains. Strains that only express LT are generally less pathogenic [26]. The NCCP15740 genome had genes encoding both STh and LT enterotoxin types. The presence of both STh and LT was common in O6 strains (21 out of 24).

The genomes of ETEC strains harbor genes encoding more than one type of CF [27]. According to a previous study [8], the CS3/CS21 genes are the most prevalent CF genes in Korean isolates, and CS3-CS21-CS1/PCF071 (15/64) and CS2-CS3-CS21 (13/64) are the most frequent CF genes in ST171. In contrast, NCCP15740 had CS2/CS3 genes that were only observed in two ST171 isolates. However, 20 out of 24 O6 strains carried CS2/CS3/CS21 genes, the major CF genes in ST171, even though they had different MLST types.

Based on the virulence factor investigation, NCCP15740 carried fewer virulence factors (59.4%) than the average number of virulence factors (62.0%) in the strains used in this study. In particular, *flgD* and *flgE* were not present in NCCP15740, but were detected in all of the reference strains. With respect to toxins, enterotoxin-related genes (*entA*, *entB*, *entC*, and *entD*) [28] were present in all of the O6 strains, including NCCP15740, whereas alpha-hemolysin-related genes (*hlyA*, *hlyB*, *hlyC*, and *hlyD*) [29] were only present in *E. coli* ATCC 25922. Alpha-hemolysin is a major virulence factor in ETEC, Shiga toxin-producing *E. coli*, and enteropathogenic strains and is thought to be acquired by horizontal gene transfer via conjugative plasmids [30].

Interestingly, none of the nonclassical virulence genes were detected in the NCCP15740 genome, and only three nonclassical virulence genes, i.e., *etpA*, *irp2*, and *tia*, were detected in other O6 strains. The O6 reference strains were isolated from patients in the USA, but the nonclassical virulence gene profiles were quite different from those of South American isolates. The *eatA*, *irp2*, and *fyuA* genes were the most prevalent in Colombian and Chilean ETEC strains [25], but none of the genes were detected in O6 strains. In addition, the *tia* and *leoA* genes were less frequent in Bolivia [31], Chile [32], Guatemala, and Mexico [33], although 11 out of 24 O6 strains had the *tia* gene.

## Future directions

In summary, NCCP15740, representing the major type of ETEC in Korea, appeared to belong to the O6 serotype and ST4. Unlike other ST4 strains, NCCP15740 did not carry the CS21 gene. Moreover, the strain harbored fewer classical virulence factors than the O6 reference strains and did not contain any nonclassical virulence factors. These results provided important insights into the development of ETEC vaccine candidates. However, because the results were obtained from in silico analyses, experimental confirmation of the results is required.

## Additional file

**Additional file 1.** Virulence factor profiles of NCCP15740 and reference strains

## Abbreviations

BSR: BLAST score ratio
CDS: coding DNA sequences
CF: colonization factor
ETEC: enterotoxigenic *Escherichia coli*
LT: heat-labile toxin
STh: heat-stable toxin
MLST: multilocus sequence typing
NCCP: national culture collection for pathogens
RAST: rapid annotation using subsystem technology
str.: strain
