# Supplementary material for: Comparative genomic analysis and characteristics of NCCP15740, the major type of enterotoxigenic Escherichia coli in Korea
Source: Gut Pathog. 2017 Sep 20;9:55. doi: 10.1186/s13099-017-0204-y (PMC5607484; doi:10.1186/s13099-017-0204-y)

Virulence	Category	Subcategory
ibeB	Adherence	CFA/I fimbriae
ibeC	Adherence	CFA/I fimbriae
eaeH	Adherence	EaeH
etpA	Adherence	EtpA
focC	Adherence	F1C fimbriae
focD	Adherence	F1C fimbriae
focF	Adherence	F1C fimbriae
focG	Adherence	F1C fimbriae
focH	Adherence	F1C fimbriae
focI	Adherence	F1C fimbriae
htpB	Adherence	Hsp60
papA	Adherence	P fimbriae
papC	Adherence	P fimbriae
papD	Adherence	P fimbriae
papE	Adherence	P fimbriae
papF	Adherence	P fimbriae
papG	Adherence	P fimbriae
papH	Adherence	P fimbriae
papI	Adherence	P fimbriae
papK	Adherence	P fimbriae
papX	Adherence	P fimbriae
sfaB	Adherence	S fimbriae
sfaC	Adherence	S fimbriae
tcpC	Adherence	Toxin-coregulated pilus (type IVB pilus)
fimB	Adherence	Type I fimbriae
fimE	Adherence	Type I fimbriae
fimG	Adherence	Type I fimbriae
wbfV/wcvB	Antiphagocytosis	Capsular polysaccharide
wbjD/wecB	Antiphagocytosis	Capsular polysaccharide
agn43	Autotransporter	Antigen 43, AIDA-I type
cah	Autotransporter	Cah, AIDA-I type
cdiA	Autotransporter	Contact-dependent inhibition CDI system
cdiB	Autotransporter	Contact-dependent inhibition CDI system
ehaB	Autotransporter	EhaB, AIDA-I type
sat	Autotransporter	Sat
upaG/ehaG	Autotransporter	UpaG adhesin, trimeric AT
upaH	Autotransporter	UpaH, AIDA-I type
vat	Autotransporter	Vacuolating autotransporter gene
rmlA	Cell surface components	GPL locus
rmlB	Cell surface components	GPL locus
gmhA/lpcA	Endotoxin	LOS
rfaD	Endotoxin	LOS
rfaE	Endotoxin	LOS
csgA	Fimbrial adherence determinants	Agf/Csg
csgB	Fimbrial adherence determinants	Agf/Csg
csgC	Fimbrial adherence determinants	Agf/Csg
csgD	Fimbrial adherence determinants	Agf/Csg
csgE	Fimbrial adherence determinants	Agf/Csg
csgF	Fimbrial adherence determinants	Agf/Csg
csgG	Fimbrial adherence determinants	Agf/Csg
fimA	Fimbrial adherence determinants	Fim
fimC	Fimbrial adherence determinants	Fim
fimD	Fimbrial adherence determinants	Fim
fimF	Fimbrial adherence determinants	Fim
fimH	Fimbrial adherence determinants	Fim
fimI	Fimbrial adherence determinants	Fim
fimZ	Fimbrial adherence determinants	Fim
stcB	Fimbrial adherence determinants	Stc
stcC	Fimbrial adherence determinants	Stc
galU	Host immune evasion	Exopolysaccharide
mrsA/glmM	Host immune evasion	Exopolysaccharide
pgi	Host immune evasion	Exopolysaccharide
kdsA	Immune evasion	LPS
lpxA	Immune evasion	LPS
lpxC	Immune evasion	LPS
cheB	Invasion	Flagella
cheD	Invasion	Flagella
cheR	Invasion	Flagella
cheW	Invasion	Flagella
cheZ	Invasion	Flagella
tia	Invasion	Tia/Hek
hemE	Iron and heme acquisition	Heme biosynthesis
hemL	Iron and heme acquisition	Heme biosynthesis
hemN	Iron and heme acquisition	Heme biosynthesis
iucA	Iron uptake	Aerobactin synthesis
iucB	Iron uptake	Aerobactin synthesis
iucC	Iron uptake	Aerobactin synthesis
iucD	Iron uptake	Aerobactin synthesis
iutA	Iron uptake	Aerobactin transport
entE	Iron uptake	Enterobactin synthesis
entF	Iron uptake	Enterobactin synthesis
fepA	Iron uptake	Enterobactin transport
fepB	Iron uptake	Enterobactin transport
fepC	Iron uptake	Enterobactin transport
fepD	Iron uptake	Enterobactin transport
fepG	Iron uptake	Enterobactin transport
hbp2	Iron uptake	Haemoglobin binding protein
chuA	Iron uptake	Hemin uptake
chuS	Iron uptake	Hemin uptake
chuT	Iron uptake	Hemin uptake
chuU	Iron uptake	Hemin uptake
chuW	Iron uptake	Hemin uptake
chuX	Iron uptake	Hemin uptake
chuY	Iron uptake	Hemin uptake
sitA	Iron uptake	Iron/managanease transport
sitB	Iron uptake	Iron/managanease transport
sitC	Iron uptake	Iron/managanease transport
sitD	Iron uptake	Iron/managanease transport
iroB	Iron uptake	Salmochelin synthesis and transport
iroC	Iron uptake	Salmochelin synthesis and transport
iroD	Iron uptake	Salmochelin synthesis and transport
iroE	Iron uptake	Salmochelin synthesis and transport
iroN	Iron uptake	Salmochelin synthesis and transport

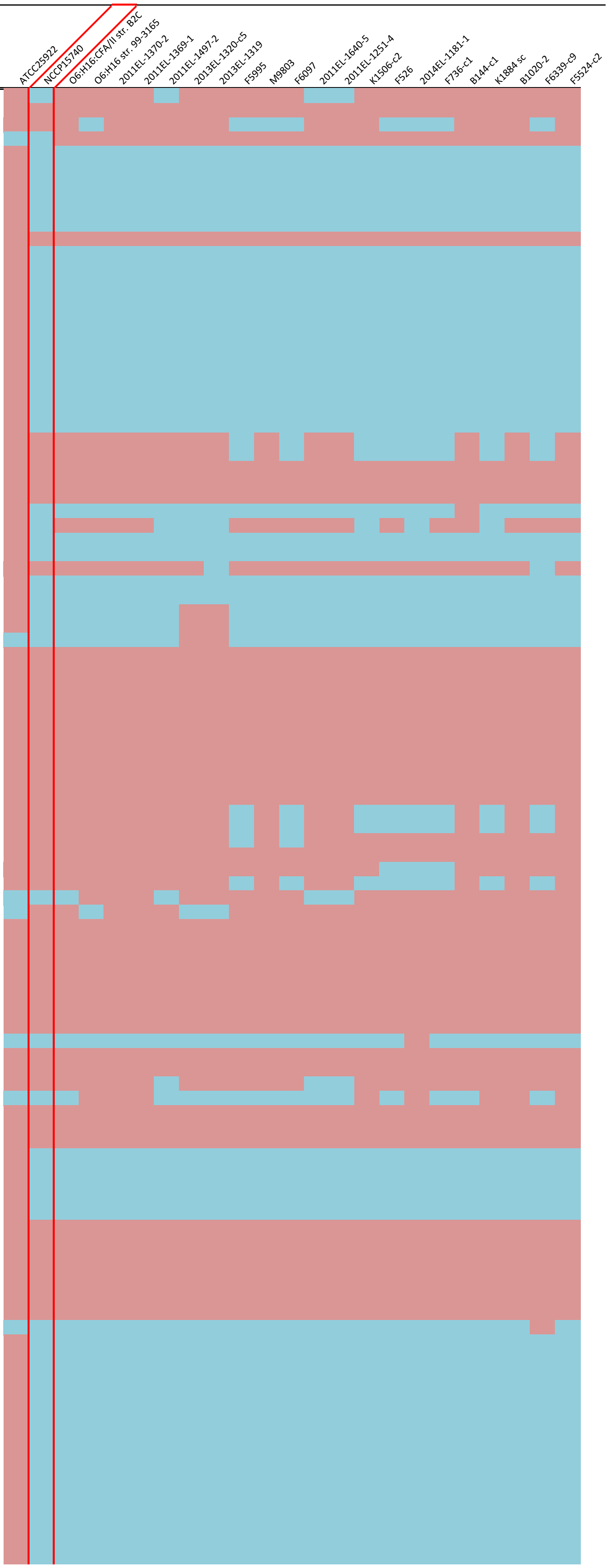

psn/fyuA	Iron uptake	Yersiniabactin
irp1	Iron uptake	Yersiniabactin siderophore
irp2	Iron uptake	Yersiniabactin siderophore
ybtA	Iron uptake	Yersiniabactin siderophore
ybtE	Iron uptake	Yersiniabactin siderophore
ybtP	Iron uptake	Yersiniabactin siderophore
ybtQ	Iron uptake	Yersiniabactin siderophore
ybtS	Iron uptake	Yersiniabactin siderophore
ybtT	Iron uptake	Yersiniabactin siderophore
ybtU	Iron uptake	Yersiniabactin siderophore
ybtX	Iron uptake	Yersiniabactin siderophore
flhF	Motility and export apparatus	Flagella
fliY	Motility and export apparatus	Flagella
motA	Motility and export apparatus	Flagella
motB	Motility and export apparatus	Flagella
espL1	Non-LEE encoded TTSS effectors	EspL1
espL4	Non-LEE encoded TTSS effectors	EspL4
espR1	Non-LEE encoded TTSS effectors	EspR1
espX1	Non-LEE encoded TTSS effectors	EspX1
espX4	Non-LEE encoded TTSS effectors	EspX4
espX5	Non-LEE encoded TTSS effectors	EspX5
sinH	Nonfimbrial adherence determinants	SinH
tuf	Other adhesion-related proteins	EF-Tu
msbB2	Others	MsbB2
virK	Others	VirK
pic	Protease	Pic
luxS	Quorum sensing	Autoinducer-2
rpoS	Regulation	Alternative sigma factor RpoS
csrA	Regulation	Carbon storage regulator A
cheA	Regulation	CheA/CheY
cheY	Regulation	CheA/CheY
aggR	Regulator	AggR
flgA	Secretion system	Flagella (cluster I)
flgB	Secretion system	Flagella (cluster I)
flgC	Secretion system	Flagella (cluster I)
flgD	Secretion system	Flagella (cluster I)
flgE	Secretion system	Flagella (cluster I)
flgF	Secretion system	Flagella (cluster I)
flgG	Secretion system	Flagella (cluster I)
flgH	Secretion system	Flagella (cluster I)
flgI	Secretion system	Flagella (cluster I)
flgJ	Secretion system	Flagella (cluster I)
flgK	Secretion system	Flagella (cluster I)
flgL	Secretion system	Flagella (cluster I)
flgM	Secretion system	Flagella (cluster I)
flgN	Secretion system	Flagella (cluster I)
flhA	Secretion system	Flagella (cluster I)
flhB	Secretion system	Flagella (cluster I)
flhC	Secretion system	Flagella (cluster I)
flhD	Secretion system	Flagella (cluster I)
flhE	Secretion system	Flagella (cluster I)
fliA	Secretion system	Flagella (cluster I)
fliD	Secretion system	Flagella (cluster I)
fliE	Secretion system	Flagella (cluster I)
fliF	Secretion system	Flagella (cluster I)
fliG	Secretion system	Flagella (cluster I)
fliH	Secretion system	Flagella (cluster I)
fliI	Secretion system	Flagella (cluster I)
fliJ	Secretion system	Flagella (cluster I)
fliK	Secretion system	Flagella (cluster I)
fliL	Secretion system	Flagella (cluster I)
fliM	Secretion system	Flagella (cluster I)
fliN	Secretion system	Flagella (cluster I)
fliO	Secretion system	Flagella (cluster I)
fliP	Secretion system	Flagella (cluster I)
fliQ	Secretion system	Flagella (cluster I)
fliR	Secretion system	Flagella (cluster I)
fliS	Secretion system	Flagella (cluster I)
fliT	Secretion system	Flagella (cluster I)
fliZ	Secretion system	Flagella (cluster I)
ipaH	Secretion system	Mxi-Spa TTSS effectors controlled by MxiE
ipaH2.5	Secretion system	Mxi-Spa TTSS effectors controlled by MxiE
gspC	Secretion system	T2SS (Type II secretion system)
gspD	Secretion system	T2SS (Type II secretion system)
gspE	Secretion system	T2SS (Type II secretion system)
gspF	Secretion system	T2SS (Type II secretion system)
gspJ	Secretion system	T2SS (Type II secretion system)
gspK	Secretion system	T2SS (Type II secretion system)
gspL	Secretion system	T2SS (Type II secretion system)
gspM	Secretion system	T2SS (Type II secretion system)
ompA	Stress adaptation	Pore-forming protein
entA	Toxin	Enterotoxin
entB	Toxin	Enterotoxin
entC	Toxin	Enterotoxin
entD	Toxin	Enterotoxin
hlyA	Toxins	Alpha-hemolysin
hlyB	Toxins	Alpha-hemolysin
hlyC	Toxins	Alpha-hemolysin
hlyD	Toxins	Alpha-hemolysin
usp	Toxins	Colicin-like Usp
eltA	Toxins	Heat-labile enterotoxin
hlyE/clyA	Toxins	Hemolysin/cytolysin A
phoP	Two-component system	PhoPQ
phoQ	Two-component system	PhoPQ
aatA	Type I secretion system	ABC transporter for dispersin
aatB	Type I secretion system	ABC transporter for dispersin
aatC	Type I secretion system	ABC transporter for dispersin
aatD	Type I secretion system	ABC transporter for dispersin
aatP	Type I secretion system	ABC transporter for dispersin
aec15	Type VI secretion system	ACE T6SS
aec30	Type VI secretion system	ACE T6SS
aec31	Type VI secretion system	ACE T6SS
aec32	Type VI secretion system	ACE T6SS
aec7	Type VI secretion system	ACE T6SS

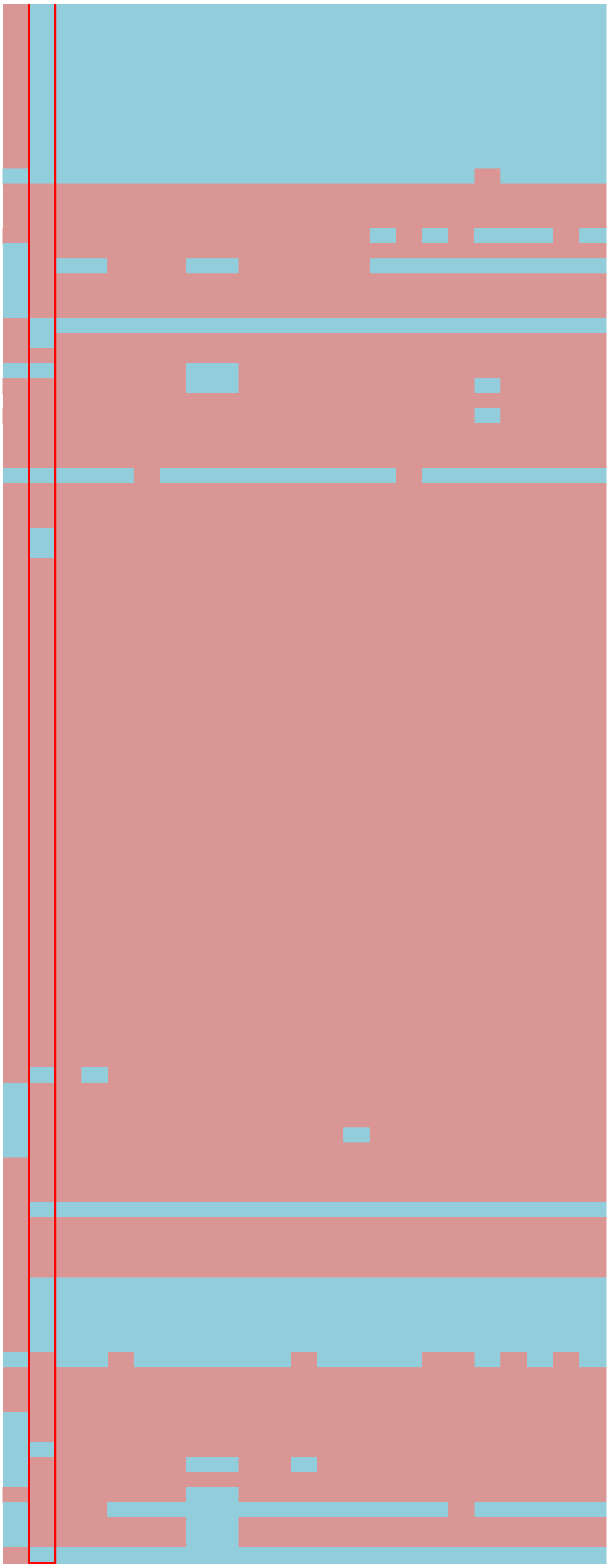

Supplement: Supplementary file 1 — Additional file 1. Virulence factor profiles of NCCP15740 and reference strains [file 13099_2017_204_MOESM1_ESM.pdf]
